# Nutritional Status and Health-Related Quality of Life among Knee and Hip Osteoarthritis Patients under Rehabilitation Care in Kuala Nerus, Terengganu, Malaysia

**DOI:** 10.5704/MOJ.2107.012

**Published:** 2021-07

**Authors:** NAA Zamri, S Harith, N Mat-Hassan, YQ Ong

**Affiliations:** 1 Faculty of Health Sciences, Universiti Sultan Zainal Abidin, Kuala Nerus, Malaysia; 2 Faculty of Medicine, Universiti Sultan Zainal Abidin, Kuala Terengganu, Malaysia

**Keywords:** nutritional status, health-related quality of life, osteoarthritis

## Abstract

**Introduction:**

The World Health Organisation (WHO) has estimated that 80% of people with osteoarthritis (OA) have movement limitations while 25% of them cannot perform their major daily activities, thus resulting in a decline of their nutritional status and quality of life (QOL). Therefore, this study aimed to compare the nutritional status and health-related quality of life (HRQOL) of OA patients between gender and age group.

**Material and Methods:**

A cross-sectional study was conducted on 131 OA patients in Rehabilitation Health Organisation, Terengganu. Socio-demographic, clinical, lifestyle histories, 24-hour dietary intake and HRQOL were assessed using a structured questionnaire.

**Results:**

Knee and/or hip OA patients recruited consisted of 19.1% of men and 80.9 % of women collectively with a mean age of 61.81 (9.28) years ranging from 38 to 83 years. The percentages of underweight, normal, overweight, and obese patients were 1.5%, 12.2%, 36.7%, and 49.6%, respectively. Further assessment of HRQOL showed that the highest mean score was obtained by the social functioning (SF) domain of 41.25 (27.16), while the mental domain scored the least mean score of 21.15 (20.92). In terms of gender breakdown, the males had significantly greater weight and height but lower body fat (BF) compared to their female counterparts, as well as a significantly higher energy, carbohydrate and protein intake. According to the age group, patients aged < 60 years had significantly greater weight, height, and BF than those aged ≥ 60 years.

**Conclusion:**

This study is an important baseline reference for proper OA management and prevention by providing crucial nutritional status and HRQOL information.

## Introduction

Osteoarthritis (OA) is a degenerative musculoskeletal disorder distinguished by gradual articular cartilage degradation, osteophyte formation, and subsequent joint space narrowing^1^. The elderly is the most susceptible population affected by this problem; as the population ages, its prevalence rises and subsequently exerts a huge economic burden on the society^2^. A study predicted that in 2010, 3.8% and 0.85% of the global population would suffer from knee and hip OA, respectively. These figures accounted for the 11th and 38th largest worldwide morbidity as measured by the disability-adjusted life years (DALYS)^3^. Accordingly, the DALYS of OA has been found to be higher than colon/rectum cancer (44th), breast cancer (47th), and Alzheimer disease (49th)^4^.

OA prevalence is increasing due to the growing size of the ageing population in developed and developing countries alike, as well as an increase in risk factors leading to the disease, particularly obesity and sedentary lifestyle^5^. Furthermore, it is associated with an increased mortality, either directly or due to its associated comorbidities. Compared to the general population, people with OA are associated with a 55% of increase in all mortality causes due to cardiovascular disease^6^. Malaysia is sharing the same experience^7^ in which Malaysian people are living longer, recording an average life expectancy level similar to other upper-middle-income countries. According to the National Health and Morbidity Survey (NHMS) 2015, the prevalence of overweight among adults in Malaysia was 30.0% while obesity recorded 17.7% of prevalence^8^. Therefore, Malaysians are associated with a high chance of increased OA prevalence due to these reasons.

In general, this disease exerts a huge impact on daily life activities, which leads to more vulnerability and dependency and thus causing a deterioration of welfare and quality of life (QOL) among the elderly^9^. WHO has reported that approximately 80% of people with OA will have limitations in their movement while 25% cannot perform their major daily activities, resulting in a significant QOL reduction^10^. Besides, alteration in body composition such as reduction of muscle mass and accumulation of body fat observed in the elderly can further influence their nutritional status^11^. Therefore, this study aims to determine the nutritional status and health-related quality of life (HRQOL) of OA patients in Kuala Nerus, Terengganu, Malaysia. Besides, the anthropometry data, dietary intake, and HRQOL information obtained were compared between the gender and age groups accordingly. It is hypothesised that there was a significant difference in nutritional status and HRQOL between the gender and age groups.

## Materials and Methods

A cross-sectional observational study utilising the universal sampling method was conducted from May 2017 to December 2018 among the knee and/or hip OA patients, which were referred from and undertaking follow-up appointments in the Rehabilitation Health Organisation (RHO) located in Kuala Nerus, Terengganu. The ethical approval was obtained from the Medical Research and Ethics Committee (NMRR-16-1937-30162) and Universiti Sultan Zainal Abidin (UniSZA) Human Research Ethics Committee (UHREC) (UniSZA C/1/uhrec/628-1). An information sheet was given to each patient together with a standard consent form before the study commencement.

The sample size consisted of 128 subjects, which was calculated using the G*Power 3.1.9.4 software according to the effect size at 0.5^7^ and confidence level at 95% using two mean formula. After considering a drop-out rate at 10%, a total of 142 patients who met the inclusion criteria were recruited in this study. The inclusion criteria required knee and/or hip OA patients from both sexes, who were diagnosed with either clinical or radiographic evidence with the disease by a medical doctor based on the International Classification of Diseases (ICD) or American College Rheumatology Criteria^12^. In contrast, the exclusion criteria detailed patients with a mental disorder or were terminally ill. Those with degenerative diseases that could affect their QOL such as cancer, heart disease, Parkinson’s disease, and more were also excluded.

This study utilised a standardised and structured questionnaire containing several parts, namely: (1) socio-demographic, (2) clinical and lifestyle histories, (3) dietary intake, and (4) Osteoarthritis Knee Hip Quality of Life (OAKHQOL) questionnaire. The patients were interviewed by the researchers, which took around 30 to 45 minutes per session. The socio-demographic part consisted of questions on respondent age, gender, race, marital status, educational level, employment status, household income, and family history of OA.

Next, the information on clinical characteristics included the type of OA, disease duration, treatment performed, and the amount and type of comorbidities. Meanwhile, the lifestyle history asked comprised of a few aspects, such as physical activity involvement; consumption of fruits, vegetables, and milk according to the Malaysian Dietary Guidelines (2010); and dietary supplements (i.e. type, frequency, and amount of intake). Dietary intake was recorded for three days (two weekdays and one day of the weekend) by using 24-hour dietary recall.

Furthermore, the HRQOL was measured by using the Malay version of OAKHQOL questionnaire, which had previously undergone the process of translation and validation and contained 31 items^13^. The questionnaire consisted of five domains with a respective Cronbach’s alpha value between 0.865 and 0.933 each, while the factor loading of each item score was above 0.65. Each item was assessed on a Likert scale ranging from zero (not at all) to 10 (a great deal). The score of each domain was multiplied by 10 and presented as a mean (i.e. divided by the number of each domain), which yielded the maximum score of 100 for each domain.

Body weight was measured twice in light indoor clothing by using an electronic scale [TANITA model UM-050] to obtain the mean value, which was recorded to the nearest 0.1kg. Meanwhile, the height was measured twice using SECA 217 stadiometer [SECA, Germany] to obtain the mean value, which was recorded to the nearest 0.1cm. In contrast, an alternative height measurement to predict the height for knee and/or hip OA patients with hunchback was utilised instead of the conventional standing height measurement. Here, their height was estimated using the demi-span using age and gender-specific demi-span equations^14^. Accordingly, demi-span was measured twice by using a measuring tape and recorded to the nearest 0.1cm on the dominant or non-paretic arm, which was taken from the finger root between the middle and ring fingers to the midpoint of the sternal notch, with the palms facing forward. Then, Body Mass Index (BMI) was calculated by dividing the weight (kg) by the squared value of height (m). The patients were then classified accordingly as underweight (< 18.5kg/m^2^), normal (18.5 to 24.9kg/m^2^), overweight (25.0 to 29.9kg/m^2^), and obese (> 30.0kg/m^2^).

Waist circumference (WC) was measured twice by using the SECA measuring tape to the nearest 0.1cm. Prior to the measurement, the patients were asked to stand erectly and breathe normally. The highest point of the hip bone and the bottom of the ribs were located by using the fingertips, whereby the measuring tape was placed between the two points and kept snug against the waist. Therefore, WC was recorded at the end of normal expiration, which was categorised accordingly as high-risk waistline > 102cm for men and > 88cm for women^15^. Meanwhile, the percentage of body fat (BF) was recorded using a weighing scale. Cut-off values of 25-31% for women and 18-25% for men were classified as acceptable, meanwhile values of >31% for women and >25% for men were considered as at risk.

The research data obtained were analysed using IBM Statistical Package for the Social Sciences (SPSS) for Windows version 22.0, whereby all data were entered, cleaned, and checked before data analysis was conducted. The normality of each variable was tested first to examine whether they met the normal distribution assumptions by using the Kolmogorov-Smirnov test. Next, non-parametric tests were used when the data were not normally distributed.

Descriptive statistics was applied to report the overall findings on the socio-demographic, clinical characteristics, lifestyle history, anthropometric measurement, dietary intake, and HRQOL elements accordingly. They were reported as either the mean (standard deviation) or median (interquartile range) for numerical variables or frequency and percentage for categorical variables.

Bivariate analysis for the parametric tests such as independent t-test was done on the data gathered, which were normally distributed. The independent t-test was used to compare the difference of two means between gender (i.e. male and female) and age category (i.e. < 60 years and ≥ 60 years) of the anthropometric measurement, dietary intake, and HRQOL parameters. The analysis was considered as significant at p < 0.05.

## Results

A total of 157 knees and/ or hip OA patients who fulfilled the inclusion criteria were approached, whereby 22 patients were then excluded due to unwillingness to participate (n = 12) and non-attendance for appointments (n = 10). Then, four patients were further excluded from the analysis due to missing values, resulting in a total of 131 (92.3%) patients who were included in the final analyses.

The mean age of the patients was 61.81 (9.28) years and ranged from 38 years to 83 years. All patients who participated in this study were of Malay descent (100.0%), whereby a majority of them aged ≥ 60 years (65.6%), were female (80.9%), married (54.9%), and had at least graduated their secondary level of education (48.8%). Similarly, more than half of the patients were unemployed (79.4%), thereby classified as housewife or pensioner, while most of them had a low household income (90.8%) with an average household income of RM 1020.39 (RM1702.56).

The mean disease duration among the patients was 4.27 (4.68) years. Most of the patients (55.8%) were diagnosed with knee and/or hip OA within one to five years, followed by 19.8% diagnosed within less than one year (i.e. at least three months). Furthermore, an almost similar proportion of patients had a family history of OA (48.1%), while the remaining had no history of OA (51.9%). An almost similar proportion of the patients had a prescribed treatment (53.4%), while the remaining were those without treatment (44.6%)The type of treatment undertaken by a majority of the patients were oral medication (44.3%).

In terms of comorbidity, the proportion of patients who had at least one (22.9%), two (26.7%), and three comorbidities (24.4%) was not too different. A total of 62.6% of the patients presented with hypertension, followed by 58.0% with hyperlipidaemia and 36.6% with diabetes mellitus. However, only 6.8% of the patients presented with other diseases, such as asthma and gastritis. Out of 131 patients, a total of 49.6%, 26.0%, 12.2%, and 12.2% were thus diagnosed with bilateral knee OA, knee and hip OA, right knee OA, and left knee OA, respectively.

A majority of the patients had a sedentary lifestyle (84.7%) and inadequate intake of vegetable (87.0%), fruit (87.8%), and milk (64.1%) accordingly. In contrast, 35.9% of the patients subscribed to supplement intake, some examples of supplements being vitamin C, vitamin B-complex, fish oil, Arabic gum, Cellmaxx© (i.e. Phenylethylamine, phycocyanin, polysaccharide, L-selectin ligand), Shaklee Vivix© (i.e. botanical beverage mix), and Pamoga juice© (i.e. beverage mix).

**Fig. 1:**
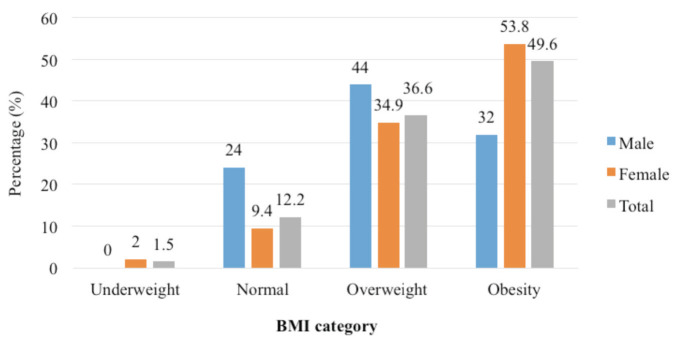
Percentage of BMI classification according to gender (n = 131).

The anthropometric assessment was performed for the patients, with the findings presented in (Table I) (gender comparison) and (Table II) (age comparison) accordingly. The mean weight, height, BMI, WC, and BF values were 72.61 (15.67) kg, 154.06 (7.26) cm, 30.51 (5.88) kg/m2, 98.89 (11.28) cm, and 41.62 (7.58)%, respectively. According to (Fig. 1), the percentage of underweight respondents was low (1.5%), while 12.2% was of normal weight. Meanwhile, the percentages of overweight (36.7%) and obese (49.6%) were highly prevalent.

**Table I:** Anthropometric measurements according to gender (n = 131)

Characteristic	Mean (SD)			Range	p-value^a^
	Male (n = 25)	Female (n = 106)	Total (n = 131)		
Weight (kg)	79.15 (18.16)	71.06 (15.67)	72.61 (15.67)	34.4 - 130.0	0.030*
Height (cm)	164.37 (5.16)	151.62 (5.28)	154.06 (7.26)	139.3 - 173.0	0.001*
BMI (kg/m2)	29.11 (5.44)	30.84 (5.95)	30.51 (5.88)	17.7 - 53.39	0.187
WC (cm)	100.74 (9.00)	98.45 (11.75)	98.89 (11.28)	63.6 - 122.7	0.363
BF (%)	32.84 (8.75)	43.69 (5.57)	41.62 (7.58)	23.3 - 58.4	0.001*

^a^Independent t-test was applied. *p < 0.05.

**Table II:** Anthropometric measurements according to age group (n=131)

Characteristic		Mean (SD)		Range	p-value^a^
	<60 years (n=45)	≥ 60 years (n=86)	Total (n=131)		
Weight (kg)	76.52 (1.13)	70.56 (16.94)	72.61 (15.67)	34.4-130.0	0.038*
Height (cm)	155.86 (6.28)	153.11 (7.59)	154.06 (7.26)	139.3-173.0	0.020*
BMI (kg/m2)	31.22 (4.17)	30.02 (6.57)	30.51 (5.88)	17.7-53.39	0.133*
WC (cm)	100.36 (10.94)	98.12 (11.44)	98.89 (11.28)	63.6-122.7	0.283
BF (%)	44.80 (5.05)	39.96 (8.16)	41.62 (7.58)	23.3-58.4	0.001*

^a^Independent t-test was applied. *p < 0.05.

**Fig. 2:**
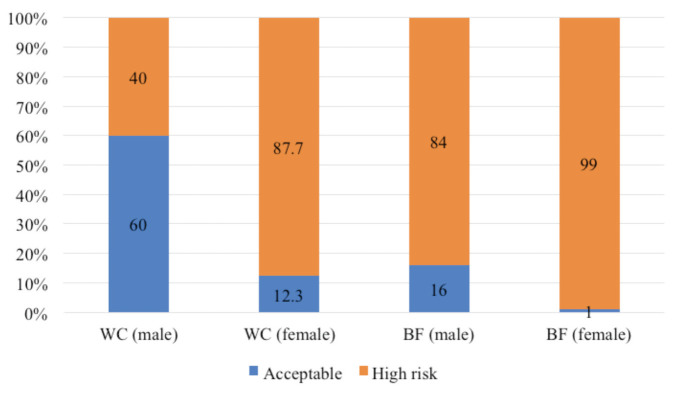
Percentage of WC and BF compared to cut-off value for male (n = 25) and female (n = 106).

Table I shows that the males have significantly greater weight (p = 0.030) and height (p = 0.001) compared to the females. However, the females showed a significantly higher BF than the males (p = 0.001). Meanwhile, BMI classification of the knee and/or hip OA patients according to gender found that the males had a higher percentage of overweight (40.0%) but a lower percentage of obese (32.0%) compared to female (34.9%, 53.8%) as shown in (Fig. 1). Next, the WC and BF measurements showed that the males had a lower percentage of high-risk group (40.0%, 84.0%), respectively compared to their female counterparts (87.7%, 99.0%) (Fig. 2).

**Fig. 3:**
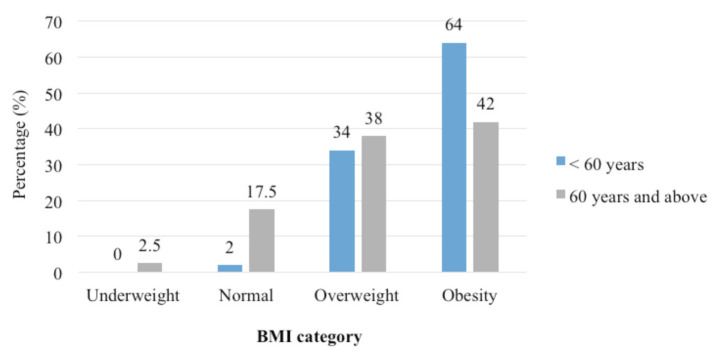
Percentage of BMI classification according to age group (n = 131).

**Fig. 4:**
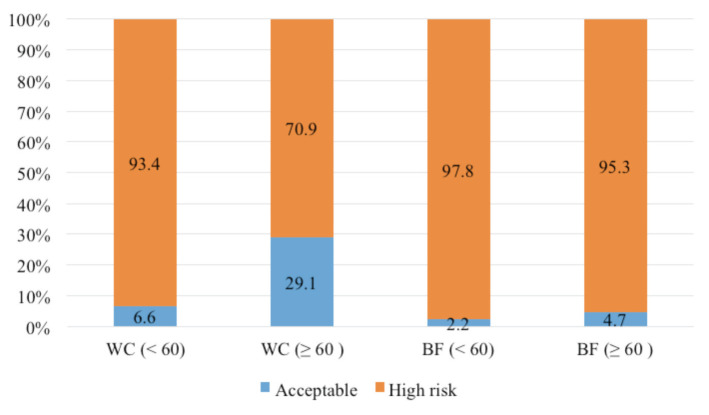
Percentage of WC and BF compared to cut-off value for patients < 60 years old (n = 45) and patient aged ≥ 60 years (n = 86).

In terms of age breakdown, patients who were < 60 years had significantly higher weight (p = 0.038), height (p = 0.030), and BF (p = 0.001) compared to those ≥ 60 years old (Table II). For the BMI classification, patients who were < 60 years old had a lower percentage of overweight (34.0%) but higher percentage of obesity (64.0%) compared to those aged ≥ 60 years (38.0%, 42.0%) (Fig. 3). Meanwhile, patients who were < 60 years had a higher risk for WC (93.4%) and BF (97.8%) compared to those aged ≥ 60 years (70.9%, 95.3%) (Fig. 4).

Table III and IV show the total energy and nutrient intakes (i.e. macronutrients and micronutrients) of the knee and/or hip OA patients according to gender and age groups, respectively. Overall, the patients consumed 1693kcal/day comprising of 227g of carbohydrate, 67g of protein, and 53g of fat.

**Table III:** Dietary intake according to gender (n = 131)

Variables		Mean (SD)		p-value^a^
	Total (n = 131)	Male (n = 25)	Female (n = 106)	
Energy (kcal)	1693.07 (610.41)	1925.24 (552.67)	1638.32 (612.89)	0.034*
Carbohydrate (g)	227.11 (57.57)	280.65 (62.14)	214.49 (48.75)	0.001*
Protein (g)	67.52 (20.28)	78.89 (29.42)	64.83 (16.54)	0.029*
Fat (g)	53.84 (21.31)	61.75 (31.06)	51.97 (17.99)	0.141
Dietary Fibre (g)	2.80 (1.78)	2.41 (1.50)	2.92 (1.83)	0.194
Sodium (mg)	1975.99 (968.58)	1923.75 (832.36)	1988.31 (1001.17)	0.766
Potassium (mg)	1448.92 (414.01)	1549.68 (552.05)	1425.16 (373.59)	0.293
Calcium (mg)	432.07 (219.55)	425.74 (267.59)	433.57 (208.08)	0.873
Cholesterol (mg)	159.50 (102.57)	193.90 (167.63)	151.39 (79.09)	0.227
Vitamin A (RE)	739.43 (437.78)	737.29 (565.89)	739.93 (405.07)	0.979
Vitamin D (μg)	8.22 (92.17)	0.04 (0.21)	10.15 (102.46)	0.624
Vitamin E (mg)	4.15 (1.34)	4.09 (1.41)	4.16 (1.32)	0.799
Vitamin K (μg)	18.44 (19.15)	14.98 (16.77)	19.25 (19.65)	0.318
Vitamin C (mg)	62.37 (45.55)	46.13 (34.49)	66.20 (47.11)	0.047*

^a^Independent t-test was applied. *p < 0.05.

**Table IV:** Dietary intake according to age group (n = 131)

Variables		Mean (SD)		p-value^a^
	Total (n=131)	<60 years (n=45)	≥ 60 years (n = 86)	
Energy (kcal)	1693.07 (610.41)	1762.36 (434.58)	1656.82 (684.27)	0.349
Carbohydrate (g)	227.11 (57.57)	229.97 (52.73)	225.62 (60.19)	0.683
Protein (g)	67.52 (20.28)	71.78 (23.56)	65.28 (18.09)	0.082
Fat (g)	53.84 (21.31)	61.37 (20.45)	49.90 (27.80)	0.003*
Dietary Fibre (g)	2.80 (1.78)	2.77 (1.65)	2.85 (1.85)	0.804
Sodium (mg)	1975.99 (968.58)	2021.34 (895.04)	1952.26 (1009.19)	0.700
Potassium (mg)	1448.92 (414.01)	1478.15 (409.17)	1433.63 (418.09)	0.561
Calcium (mg)	432.07 (219.55)	457.31 (226.71)	418.87 (215.87)	0.343
Cholesterol (mg)	159.50 (102.57)	181.66 (11.93)	147.91 (94.74)	0.074
Vitamin A (RE)	739.43 (437.78)	955.42 (492.45)	626.41 (360.50)	0.001*
Vitamin D (μg)	8.22 (92.17)	23.64 (157.25)	0.15 (0.82)	0.167
Vitamin E (mg)	4.15 (1.34)	4.49 (1.49)	3.97 (1.22)	0.032*
Vitamin K (μg)	18.44 919.15)	19.42(20.07)	19.92 (18.75)	0.673
Vitamin C (mg)	62.37 (45.55)	66.37 (54.96)	60.27 (36.48)	0.468

^a^Independent t-test was applied. *p < 0.05.

Considering Table III, the male respondents consumed significantly more energy (p = 0.034), carbohydrate (p = 0.001), and protein (p = 0.029) but less vitamin C (p = 0.047) than the females. However, no significant mean difference was seen in terms of fat, dietary fibre, sodium, potassium, calcium, cholesterol, and vitamin A, D, and K intakes between the male and female respondents.

Besides, a significant mean difference was observed regarding fat (p = 0.003), vitamin A (p = 0.001), and vitamin E intakes (p = 0.032) between patients aged < 60 years and ≥ 60 years (Table IV). Patients aged < 60 years consumed more fat, vitamin A, and vitamin E compared to their counterparts (aged ≥ 60 years). In contrast, no significant mean differences were observed for the remaining nutrient intakes between the age groups.

The HRQOL score according to the domains is shown in (Table V) (gender comparison) and VI (age group comparison) accordingly. The scores were standardised on a scale from 0 (best possible QOL) to 100 (worst possible QOL). Overall, SF domain recorded the highest value of 41.25 (27.16), while mental domain recorded the lowest score of 21.15 (20.92). According to gender as shown in (Table V), the males reported the highest score in social functioning at 43.33 (30.99) while the females obtained the highest score in the physical domain 41.62 (22.89). However, no significant difference in the HRQOL domains was observed in both genders.

**Table V:** HRQOL according to gender by using OAKHQOL questionnaire (n = 131)

Mean (SD)		Mean (SD)		p-value^a^
	Total (n=131)	Male (n=25)	Female (n=106)	
Physical	41.6 (22.81)	38.69 (22.81)	41.62 (22.89)	0.566
Mental	21.15 (20.92)	15.95 (12.71)	22.37 (21.86)	0.168
Pain	38.78 (22.05)	32.00 (20.26)	40.33 (22.25)	0.097
SF	41.25 (27.16)	43.33 (30.99)	40.75 (26.32)	0.671
SS	21.32 (18.70)	19.93 (21.70)	21.89 (17.99)	0.480

^a^Independent t-test; SF-Social Functioning; SS-Social Support

In terms of age, the patients aged < 60 years were more affected in the pain domain with 39.67 (23.29), while those aged 60 years and above were more affected in the physical domain 43.27 (25.40). Next, a significantly higher score in the mental domain was observed in the patients aged < 60 years compared to their counterparts (p = 0.028), while those aged ≥ 60 years scored higher in the SF domain (p = 0.044) (Table VI).

**Table VI:** HRQOL according to age group by using OAKHQOL questionnaire (n = 131)

Domain		Mean (SD)		p-value^a^
	Total (=131)	<60 years (n=45)	≥ 60 years (n = 106)	
Physical	41.06 (22.81)	36.84 (16.23)	43.27 (25.40)	0.081
Mental	21.15 (20.92)	26.69 (25.68)	18.24 (17.41)	0.028*
Pain	38.78 (22.05)	39.67 (23.29)	38.31 (21.49)	0.740
SF	41.25 (27.16)	34.67 (27.36)	44.68 (26.57)	0.044*
SS	21.32 (18.70)	21.11 (16.920)	21.43 (16.92)	0.926

^a^Independent t-test; SF-Social Functioning; SS-Social Support

## Discussion

The present study found that the mean age of the patients was 61.81 (9.28) years, which was relatively younger compared to a previous local study^16^ and other international studies^11,17^. A majority of the patients involved in this study were 60 years old or older, which was similar to a previous study^17^. Therefore, OA is strongly associated with ageing, whereby a scoping review has reported that subjects aged 50 years and above are more vulnerable to OA compared to those younger than 50 years old^18^. These findings are supported by a previous research report that the increasing OA prevalence with age may be caused by biological changes. They render a joint less capable to cope with adversities, such as cartilage thinning, weak muscle strength, poor proprioception, and oxidative damage^19^.

Besides, this current study found that a majority of the patients involved were females (80.9%), which was in agreement with previous studies^11,20-22^. Females are usually associated with a higher prevalence and more severe case of OA due to menopausal effect. The significant increase of OA in female around the time of menopause has led to multiple investigations of the hormonal effect on its pathophysiology. However, the results of the effect of oestrogen therapy have been conflicting as oestrogen usage is linked to a healthy lifestyle and osteoporosis, which can lower the risk of OA. Besides, females may have a reduced volume of knee cartilage than the males but it is not clear if this can contribute to an accelerated cartilage loss^20^.

Furthermore, the present study found that most of the patients involved were unemployed (79.4%) consisting of housewives and pensioners, as well as having a low household income (90.8%). These findings are in agreement with a previous study conducted in India^23^, which has reported that the socioeconomic status can be measured in terms of the income, wealth, poverty, education, occupation, and area-level measures^24^. Similarly, a study by Callahan *et al* (2011)^25^ has found that both education and socioeconomic status are independently associated with knee OA, whereas Robbel *et al* (2014)^23^ has stated that low socioeconomic status comprising of low educational attainment, occupational status, and income may pose an additional risk factor for the disease development.

Moreover, the present study found that the mean disease duration was 4.27 (4.68) years. This is comparable to a local study^16^ and other international studies^26^ accordingly. Besides, it was found that a majority of the patients had been diagnosed with knee and/or hip OA within one to five years (55.8%), which was in agreement with Harsha Kumar *et al* (2015)^22^. Meanwhile, most of the patients in this study had two comorbidities (26.7%), which paralleled previous studies^16,17,22^. Additionally, a majority of the patients had hypertension (62.6%), followed by hyperlipidaemia (58.0%) and diabetes mellitus (36.6%), which reflected the prevalence of these three diseases among the adults in Malaysia^27-29^. A meta-analysis study had shown a significant relationship between hypertension and knee OA (i.e. radiographic and symptomatic)^30^.

The present study found that a majority of the patients had a sedentary lifestyle (84.7%) and inadequate intake of vegetable (87.0%), fruit (87.8%), and milk (64.1%). Most reported that they had limited movements or were sedentary due to knee pain, which was noted in a previous study that correlated a longer sedentary behaviour with chronic knee pain. A sedentary behaviour exceeding 10 hrs/day has been noted to be significantly associated with chronic knee pain. However, participants with high levels of physical activity were less likely to suffer from chronic knee pain, while women with over 10 daily hrs of sedentary behaviour and high level of physical activity were more likely to have chronic knee pain. The researcher has also reported a significant association between chronic knee pain and obese (≥ 30.0kg/m^2^) individuals^31^.

Accordingly, the present study showed a low intake of vegetables and fruits among the patients, which was in agreement with a few previous studies. A majority of the patients also reported the inadequate intake in their daily lives was due to food availability and chewing difficulties, especially in the elderly group. A recent cross-sectional study from the Korean National Health and Nutrition Examination Survey has revealed the higher intake of fruits and vegetables to be significantly associated with a lower prevalence of knee pain in older adults with knee OA^32^. Meanwhile, another study from India has reported that low fruit consumption is associated with a high prevalence of knee OA^33^. Additionally, a researcher has shown that dietary fruits, especially berries, are a rich source of several phytochemicals and nutrients, which may explain much of their physiological effects as antioxidants and anti-inflammatory agents^34^.

The present study also found that low milk intake among the patients was due to the high cost of milk purchase, whereby the low intake could be supported by a few previous studies. A previous study conducted among the Dutch population has indicated that a higher intake of full-fat dairy and Dutch cheese products as opposed to milk is associated with a lower presence of knee OA^35^. Besides, a prospective study by Lu and colleagues has included 2,148 men and women participants in the Osteoarthritis Initiative (OAI) in which a baseline dietary assessment is done, enquiring the usual frequency of milk, yoghurt, and cheese intake over the past 12 months. Then, OA progression over 48 months of follow-up was evaluated as a change in quantitative joint space width (JSW) between the medial femur and tibia of the knee based on plain radiographs. As a result, the main finding noted that a greater milk consumption was associated with reduced joint space narrowing in women but not in men. Therefore, the researchers concluded that women who were “milk drinkers” would experience 4-year decrease in JSW, specifically from 0.09 to 0.12 mm, than the non-milk drinkers. However, a more frequent cheese intake is associated with a greater joint space narrowing in women alone, while no associations are seen with yoghurt intake^36^.

The present study found that the mean BMI was 29.11 (5.44) kg/m^2^, which was in agreement with a local study^16^ but a bit lower compared to Rogers and Wilder^37^. A majority of the patients was categorised as obese (49.6%). A previous study has reported that high BMI is a risk factor of knee OA^38^, whereas obesity (i.e. defined as BMI ≥ 30kg/m^2^) and abdominal obesity are strongly related to radiographic knee OA. Moreover, it has been noted that every 5kg of body weight gained increases the risk of knee OA by 36%^39^. Therefore, healthcare professionals should take account of a possible weight reduction in planning the treatment of knee OA whenever a patient is significantly overweight.

Besides, the WC variable in the present study revealed that a majority of the male respondents was categorised as acceptable (60.0%), while most of the females were categorised as the high-risk group (87.7%). This is similar with a previous study^40^, which has also reported central obesity as a key factor in metabolic syndrome (MetS). The relationship between OA and MetS or its components has been evaluated accordingly by Engstrom *et al* (2009)^41^, whereby it is found that MetS is associated with an increased incidence of knee OA in a Western population after adjustments are made for the age, sex, and social factors^41^.

Overall, the present study found that the most affected HRQOL parameter among the patients was social functioning, which was similar to a previous study^42^. In contrast, the least affected HRQOL parameter was mental health domain, which differed from Ouedraogo *et al* (2014)^42^ as the study reported the domain to be most affected by using the SF-36 questionnaire. The social functioning domain was the most affected parameter in the present study due to ageing and disease severity among the patients, who reported their social functioning to be less functional compared to pre-diagnosis of knee and/or hip OA. Therefore, they preferred to isolate themselves from other people due to the disease severity. Moreover, the present findings are consistent with a previous study that had reported poorer social functioning to be related to more depressive symptoms, but both poorer social and physical functioning predicted worse perceived in health^43^.

In the present study, the mental health domain was the least affected parameter in the HRQOL, which was similar to a local study^16^ despite the use of a different instrument, namely the SF-36 Questionnaire. The mental health domain was found to be the least affected due to better coping mechanisms and adaptation to the chronic disease. A previous study has found patients with knee OA have various coping mechanisms and result in less pain and better mood compared to those with rheumatoid arthritis^44^. In contrast, the previous studies by Mahmoud *et al* (2019) and Ouedraogo *et al* (2014)^26,42^ have reported that the pain domain is the least affected parameter in HRQOL.

The present study found that the male respondents had higher body weight and height but lower BF compared to the females. The present findings on BF were consistent with those of a previous study by Blaak (2001)^45^. Besides, for BMI categorisation, the males had a lower percentage (32.0%) of obesity status than the females (53.8%), which was parallel to a previous study by Kim *et al* (2010)^46^. Meanwhile, male WC showed a lower (40.0%) percentage of high risk compared to the female WC (87.7%)^47^. Similarly, dietary intake showed significant differences for energy, carbohydrate, protein, and vitamin C between male and female in this study. Since a limited number of studies are done on dietary intake, a comparison is not possible. For HRQOL, no significant difference was seen for all domains between male and female, regardless. The present study concluded with the results that females had a higher percentage of body fat, obesity status, and high risk of WC.

Similarly, the present study found that patients aged < 60 years had higher weight, height, and BF compared to those aged ≥ 60 years. This finding is inconsistent with a previous study;^48^ although the subject of the study is of a healthy population, the researcher has reported that a decrease in fat-free mass and increase in BF percentage are observed with ageing. In contrast, the present findings on WC showed inconsistencies with the previous study, which reported that abdominal obesity increases with ageing^49^. For dietary intake, patients aged < 60 years consumed more fat and vitamin A and E compared to those aged ≥ 60 years. This may be attributed to fat malabsorption commonly present among those aged ≥ 60 years^50^.

For HRQOL, the present study found that patients aged < 60 years had a higher mental score and a lower social functioning score compared to those aged ≥ 60 years. This indicated that patients aged ≥ 60 years had better mental health but poorer social functioning, which was similar to^16^. Therefore, it could be concluded that the high prevalence of knee and/or hip OA patients in the present study was due to the high percentage of BF, obesity status, and high risk for WC among patients aged < 60 years. Therefore, some of the secondary prevention methods include dietary intervention and exercise, which are the basic management for individuals who are overweight or obese^51^.

This study, however, has several methodological limitations. The present study was conducted in the rehabilitation centre in Kuala Nerus, Terengganu only due to financial constraints, thus limiting generalisation of the results to other communities, which comprised of other ethnicities such as Chinese and Indians, whereby dietary intake are likely to be different. This is because Kuala Nerus had 97.0% of the Malay population, and the rest are Chinese, Indians, and other ethnicities, according to the Department of Statistics Malaysia (2010)^52^. The findings in the dietary intake of knee and/or hip OA are subject to limitations. The assessment of dietary intake in the present study using the 24-hr dietary recall maybe overreporting or underreporting of total calorie and nutrient intake. More independents variables should be considered such as biochemical data, specific physical activity data and specific rehabilitation program should be considered to get more accurate results.

## Conclusion

Most of the OA patients in this study were inactive and had an imbalance of dietary intake, resulting in a majority of them being overweight or obese and had a high percentage of BF and adiposity fat, which was measured as the WC. In terms of gender breakdown, the males had significantly greater weight and height but lower BF compared to their female counterparts, as well as a significantly higher energy, carbohydrate, and protein intake. However, no significant differences were found in the HRQOL between genders. Meanwhile, according to the age group, patients aged < 60 years had significantly greater weight, height, and BF than those aged ≥ 60 years. Similarly, the fat and vitamin A and E intakes were significantly lower in patients aged ≥ 60 years. Next, a significantly higher score in the mental domain was observed in the patients aged < 60 years, while those aged ≥ 60 years scored higher in the SF domain. Hence, this study is an important baseline reference for proper knee and/or hip OA management and prevention. Accordingly, healthcare professionals should take into account regarding possible weight reduction and a physically active lifestyle in order to prevent OA.
